# Automated Determination of Arterial Input Function for Dynamic Susceptibility Contrast MRI from Regions around Arteries Using Independent Component Analysis

**DOI:** 10.1155/2016/2657405

**Published:** 2016-07-28

**Authors:** Sharon Chen, Yu-Chang Tyan, Jui-Jen Lai, Chin-Ching Chang

**Affiliations:** ^1^Department of Medical Imaging and Radiological Sciences, Kaohsiung Medical University, Kaohsiung 807, Taiwan; ^2^Center for Infectious Disease and Cancer Research, Kaohsiung Medical University, Kaohsiung 807, Taiwan; ^3^Graduate Institute of Medicine, College of Medicine, Kaohsiung Medical University, Kaohsiung 807, Taiwan; ^4^Institute of Medical Science and Technology, National Sun Yat-sen University, Kaohsiung, Taiwan; ^5^Department of Medical Imaging, Kaohsiung Medical University Hospital, Kaohsiung 807, Taiwan

## Abstract

*Purpose.* Quantitative cerebral blood flow (CBF) measurement using dynamic susceptibility contrast- (DSC-) MRI requires accurate estimation of the arterial input function (AIF). The present work utilized the independent component analysis (ICA) method to determine the AIF in the regions adjacent to the middle cerebral artery (MCA) by the alleviated confounding of partial volume effect.* Materials and Methods.* A series of spin-echo EPI MR scans were performed in 10 normal subjects. All subjects received 0.2 mmol/kg Gd-DTPA contrast agent. AIFs were calculated by two methods: (1) the region of interest (ROI) selected manually and (2) weighted average of each component selected by ICA (weighted-ICA). The singular value decomposition (SVD) method was then employed to deconvolve the AIF from the tissue concentration time curve to obtain quantitative CBF values.* Results*. The CBF values calculated by the weighted-ICA method were 41.1 ± 4.9 and 22.1 ± 2.3 mL/100 g/min for cortical gray matter (GM) and deep white matter (WM) regions, respectively. The CBF values obtained based on the manual ROIs were 53.6 ± 12.0 and 27.9 ± 5.9 mL/100 g/min for the same two regions, respectively.* Conclusion.* The weighted-ICA method allowed semiautomatic and straightforward extraction of the ROI adjacent to MCA. Through eliminating the partial volume effect to minimum, the CBF thus determined may reflect more accurate physical characteristics of the T2^⁎^ signal changes induced by the contrast agent.

## 1. Introduction

Perfusion is a fundamental physiological characteristic of brain tissues that can be measured by MRI techniques. One of the MRI methods commonly applied in clinical settings for measuring cerebral blood flow (CBF) is the dynamic susceptibility contrast MRI (DSC-MRI) [1, 2]. The DSC-MRI, an exogenous contrast technique, allows rapid measurements of MRI signal change when the paramagnetic bolus agent passes through the brain tissue. DSC-MRI with high SNR has led to widespread clinical applications such as initial investigation of stroke and tumor imaging [2, 3].

High concentrations of lanthanide contrast agents (e.g., Gd-DTPA) produce significant T2 and T2^*∗*^ relaxation and cause the signal to drop by about 50% when the blood-brain barrier is intact. Vilringer et al. [4] presented a first-order model to explain the local magnetic inhomogeneity across vessels due to the induced susceptibility difference. They found that inhomogeneity occurs mainly in the regions of tissue around vessels and the magnitude of variability is inversely proportional to the square of the distance from the center of the vessel. In contrast to T1 signal enhancement, which has a short range of interaction, the T2 susceptibility effect extends beyond the vascular space, affecting much of the surrounding brain tissue [5]. Duhamel et al. [6] found that the arterial input function (AIF) determined from regions within arteries, instead of around arteries, could result in uncertainty in the estimated mean transit time (MTT). One can conclude from these experiments that it is more accurate to measure AIF from regions around the artery.

AIF plays an important role in the quantification of CBF for perfusion measurements. CBF can be obtained by deconvolving AIF from the measured concentration time curve of tissue with dilution theory equation [7, 8]. How and where the AIF was determined has been one of the key aspects in calculating perfusion parameters. While obtaining the local AIF for each imaging voxel is difficult, a surrogate AIF is usually derived from one of the major arteries, for example, the middle cerebral artery (MCA) [5, 6]. Practically, the AIF is commonly determined by manual selection of regions of interest (ROIs) surrounding large arteries [5, 9]. Compared with that derived from regions within large vessels, AIF derived from tissues adjacent to vessels avoids flow artifacts and possible signal saturations while examining T2 changes resulting from the contrast agent passage. In addition, it provides more accurate CBF quantification since the relaxivity constant embedded in the concentration time curves of the AIF would be closer to that of the tissue. Next to the understanding of AIF characteristics, postprocessing of signal extraction is very important. Van Osch et al. [10, 11] have recently used calibration curves incorporated with partial volume correction algorithm by selecting manually a region covering the tissue around the internal carotid artery, which showed improved reproducibility of AIF determination. However, in their study, AIF was obtained from blood signals and the vessel was required to be parallel to the main magnetic field because the phase shift is linear along with the cross session of the vessel. On the other hand, partial volume correction factor is also a way to eliminate the partial volume effect by scaling the tail of concentration time curve of artery and vein [12, 13]. For AIF determination by automatic method of ROI selection, several research groups [14, 15] proposed methods by setting criteria related to the characteristics of the dynamic signal/concentration time curves, such as full width at half maximum (FWHM), the maximum concentration (MC), time-to-peak (TTP), and arrival time (AT). Although the processes were automatic, these methods were limited by the lack of biophysical meanings for criterion selected because the MRI signal was combined with both T1 and T2 effects, which could vary with different imaging systems, protocols, and patients or subjects.

Manual ROI technique and criterion ROI method are subjective and cumbersome in resolving the confounding signal which is mixed with various tissue components around vessels. Therefore, there has been thriving use of numerical method to automatically segment the ROI. The statistical methods that examine the difference in signal characteristics are appropriate for solving the problem of signal mixture. Martel et al. [16, 17] applied factor analysis (FA) technique combined with principle component analysis (PCA) to remove much of the random noise contamination when extracting the vessel factor image with the signal intensity curve for 107 patients with acute stroke. However, additional assumptions with* a priori* knowledge were needed to yield factors with physiological significance. Murase et al. [18] presented the fuzzy *c*-means (FCM) method for determining AIF within the mask around the internal carotid artery. This method was complex in mask decision and had difficulty defining the number of clusters. Moreover, determining the fuzzy rules of the cluster was subjective and difficult.

A blind source separation method such as independent component analysis (ICA), which decomposes the mixture signals into basic components, can be employed to extract the signal of interest. ICA is a model-free multivariate statistical method that has been employed to identify pixels that have a common underlying time-response behaviors involving the spatially independent cortical activation areas in functional MRI (fMRI) [19–25]. It had also been used as a segmentation technique to visualize the different hemodynamic compartments [26, 27] and to remove the confounding signals from large vessels to improve images with significantly less artifacts [28]. The present work proposed using ICA to define regions around the artery and to determine an accurate AIF from the regions. AIFs were also obtained from ROIs that were drawn manually and from the regions within arteries as determined by ICA. CBFs were then calculated by these AIFs for comparison. The resultant CBF from ICA-based method got closer values 41.1 ± 4.9 mL/100 g/min and 22.1 ± 2.3 mL/100 g/min for gray matter and white matter, compared to the standard values of nuclear medicine, 43.1 mL/100 g/min and 21.3 mL/100 g/min, respectively [29].

## 2. Materials and Methods

### 2.1. Simulation Experiment

**Figure 1:**
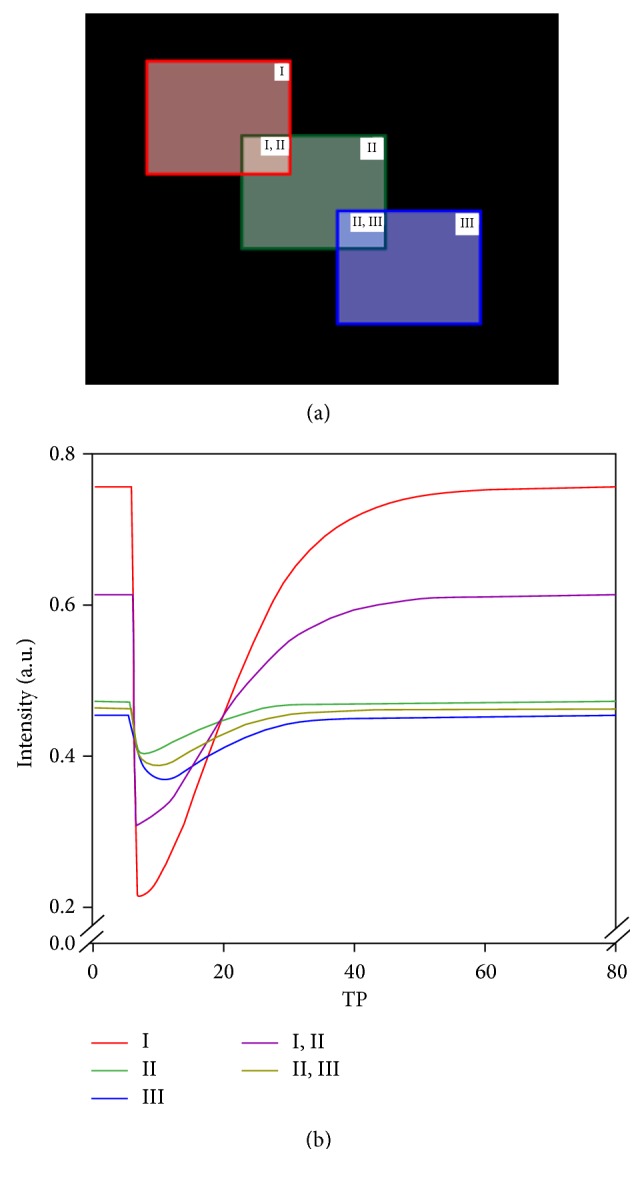
(a) The physiological signals for artery (I), surrounding tissue (II), and tissue (III) are simulated in three blocks (in red, green, and blue, resp.). The partial volume fractions between (I, II) and (II, III) were 0.5. (b) The generated raw signals were calculated from Kiselev's approach [30].

The purpose of simulation experiment is to evaluate the performance of image segmentation by ICA. Three squared blocks (each with 81 pixels) representing the artery (in red), artery-surrounding tissue (in green), and tissue (in blue), respectively, are shown in Figure 1(a). The signals mimicking the contrast agent effect on vessel and parenchyma are calculated from Kiselev's approach [30]. In his approach, he utilized Pade approximation to bridge the deviance between theoretically known limits with the account for the results of the Monte Carlo simulation [5]. The deviance caused by the effect of contrast agent is due to the local field inhomogeneity around the vessel and capillary. The force of inhomogeneous field around the vessel is the susceptibility difference between inside and outside vessel, which is the static dephasing regime (SDR) effect. Another force causing the field inhomogeneity around capillary is due to the proton diffusing out toward the tissue, which is called the diffusional narrowing regime (DNR) effect.

In our simulation, we simply assumed the signal of artery-surrounding tissue is affected only by the SDR effect and that of tissue is affected by the combination of the DNR and SDR effects. The input bolus of artery was given as

(1) $$\begin{array}{c} C_{a}\left(\;t\;\right)=\frac{C_{max}\left(t/t_{0}\right)}{\exp\left(-t/t_{0}+1\right)}, \end{array}$$

where *C*_max_ = 3 mM and *t*_0_ = 7 ms [30]. The concentration of contrast agent in blood in the studied tissue was obtained through the calculation of dilution theory (mean transit time was assumed as 2.6 s). The inhomogeneity of field around vessels was related to several factors, such as magnetic field (*B*_0_ = 1.5 T), pulse sequence (Gradient Echo with TE = 45 ms), blood volume fraction (*ζ*_*a*_ = 0.005; *ζ*_*v*_ = 0.01; *ζ*_*c*_ = 0.02), vessel radius (*ρ*_*a*_ = *ρ*_*v*_ = 100 *μ*m; *ρ*_*c*_ = 3.5 *μ*m), the magnetic susceptibility of venous blood (*χ*_0_ = 0.038 ppm), the baseline relaxation rate (*R*_2*a*_0__ = 6.21 1/s; *R*_2*v*_0__ = 13.43 1/s), and the relaxivity [30]. The relaxivity was estimated from the known parameters following the asymptotic forms in [30].

The noise-free signal time curves for the three components (I, II, and III) were converted from the simulated concentration time curves and shown in Figure 1(b). The partial volume fraction in the overlapped areas (I&II and II&III) was set to be 0.5. Various levels of noise were added to generate contrast-to-noise ratios (CNRs) ranged from 30 to 70 for the arterial signal time curve. Images of 80 time points with 1.5 s per time point were simulated for each CNR. Using ICA, 10 components were extracted and three of them were selected for each of the three signal sources. The IC maps were transferred into *z*-value maps, and the first 50 voxels with maximum *z*-values were selected. The performance of segmentation was evaluated with percent detected voxels in the ROIs with and without partial volume averaging.

### 2.2. MRI Acquisition

Eight healthy volunteers (4 males and 4 females), 30–45 years old (average = 35.5 years), participated in this study with informed consent. All experiments were performed at Chang Gung Memorial Hospital with protocols approved by the institutional review board.

A single-shot spin-echo EPI sequence was employed to perform the perfusion imaging on a 1.5 T MR scanner (Magnetom Vision, Siemens, Erlangen, Germany) with the following parameters: TR/TE = 1,500/60 ms, flip angle = 90°, field of view = 218 × 218 mm^2^, matrix size = 64 × 64, and slice thickness = 6 mm. Seven image slices with 60 time points per slice were obtained for each subject. The images of the first three time point images were discarded as dummy scans. In each perfusion measurement, the contrast agent Gd-DTPA (0.2 mmol/kg b.w., Magnevist, Schering, Berlin, Germany) was injected in the left antecubital vein using an MR-compatible injector (Spectris, Medrad Inc., Indianola, PA). The injection rate was set to be 5 mL/s and the volume of the dose was 25 mL. The time point (TP) of injection was at 7th TP of scan. Two volunteers were excluded from the evaluation due to significant motions during the DSC-MRI scan.

### 2.3. Data Processing

AIF was selected using both the manual and ICA methods. For the manual method, a region (about 30 pixels) around the MCA was singled out as the candidate of AIF calculation. As for the ICA method, the data process is described as follows.

ICA decomposed the input data into their constituent sources, according to statistical independence [31, 32]. In this technique, an unmixed matrix (*W*_*q*×*p*_) is employed to project data into its own reconstructed source (*N*_*q*×*v*_) in the *q* direction where data distribution is non-Gaussian:

(2) $$\begin{array}{c} M_{q_{0}\times v}\approx N_{q\times v}=W_{q\times p}X_{p\times v}, \end{array}$$

where *M*_*q*_0_×*v*_ is the source matrix with *q*_0_ sources and spatial size *v*; *X*_*p*×*v*_ is the observed data matrix with *p* time series; *W*_*q*×*p*_ is the unmixed matrix with *q* × *p* matrix size; *N*_*q*×*v*_ is the component matrix which is employed to approach the original component source (*M*), where *q*≦*q*_0_.

Forty constitutional IC maps (covering 99% of the eigenvalues) were generated based on tissue characteristics with which tissue had its temporal performance in the independent spatial domain. Of all IC maps, two maps of interest, namely, the artery (ICA-a) and the tissue around the artery (ICA-s), were selected according to their hemodynamic characteristics; that is, artery has early, narrow, and high peak features of responsive concentration. In order to decide a global AIF, the selected IC map was transferred into *z*-value maps, and the first 50 voxels of maximum *z*-value were used as the ROI of AIF calculation. The *z*-value is defined as *z*_*i*_ = (*x*_*i*_ − mean_IC_)/Std_IC_, where *x*_*i*_ is the voxel value of an IC image; “mean_IC_” and “Std_IC_” are the average and standard deviation over an IC map [32]. Therefore, the time course per voxel in the ROI was weighted by the *z*-value of the IC map to generate an AIF. This weighted time course embodied the tissue's response to contrast agent and facilitated deciding a more purified region with respect to the representation of tissue. That is because, in ICA calculation, the temporal behaviors of voxels having the same tissue properties were grouped into a component map because it contributed the same value to the location of anatomy. Taking this advantage, ICA selected voxels with higher weighting to calculate AIF and removed the partial volume effect. The weighted-AIF can be defined by

(3) $$\begin{array}{c} AIF=\overset{n}{\underset{i}{\sum }}Q_{i}\times S_{i}\;n=voxels\;\;in\;\;VOI, \end{array}$$

where *S*_*i*_ is the signal intensity time curve of the *i*th voxel in the ROI selected after *z*-value thresholding and region clustering in each ICA map of interest. *Q*_*i*_ is the weighting matrix of the *i*th voxel whose value is the normalized value from the IC map, and herein AIF is termed as AIF_(ICA-w)_.

Before calculating CBF, the MR signal intensity was transferred into the concentration of contrast agent [5, 30] by the following equation:

(4) $$\begin{array}{c} C\left(\;t\;\right)=\frac{-K}{TE}\ln\left(\;\frac{S\left(t\right)}{S_{0}}\;\right), \end{array}$$

where *K* is the relaxivity, which is related to the tissue type and magnetic field. For 1.5 T magnetic field, *K* is about 7.62 m/M/s; *S*(*t*) is the signal intensity at time *t*; and *S*_0_ is the baseline intensity before the contrast arrival. Afterward, the concentration time curve of candidate AIFs was fitted using the data period of 7th–25th time points with a gamma-variate function to determine the TTP, arrival time, FWHM, and peak height as the indicators of AIF for comparison of two AIFs. Later, the concentration of candidate AIFs was employed to calculate CBF using the SVD deconvolution method with adaptive thresholds [33].

## 3. Results

**Figure 2:**
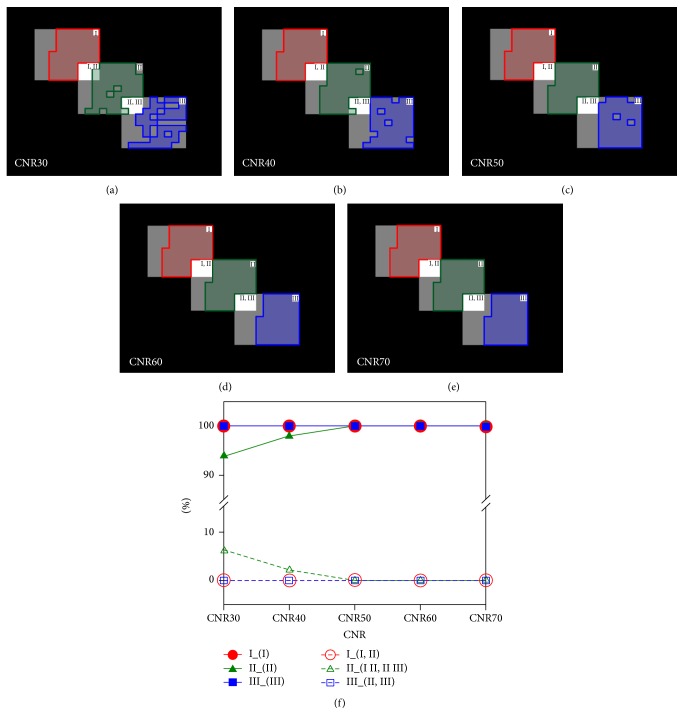
The performance of signal segmentation for three ROIs after ICA. The spatial segmentation of ROIs was listed in (a)–(e) along different CNRs. The area symbols for artery, surrounding tissue, and tissue are denoted as I, II, and III, respectively. The performance in signal segmentation is summarized in (f). The solid line denotes the true rate of selected region located in the ROI and the dash line is the false rate outside ROI.

In the simulation experiment, the performance of signal segmentation with ICA as a function of CNR was presented in Figure 2. The three IC component maps (which selected at most 50 pixels for each ROI) corresponding to each of the three signal sources were demonstrated in Figures 2(a)–2(e) for different CNR levels. The alleviation of partial volume effect (in I&II and II&III areas) was observed as CNR increase. The segmentation performance at various CNR was summarized in Figure 2(f). Comparing to surrounding tissue, the localization of signal for artery and tissue was fully achieved. The partial volume effect affected the signal decomposition, especially for regions containing boundary at low CNR condition. However, the segmentation of surrounding tissue was quite accurate, even at low CNR level (>90% accuracy).

**Figure 3:**
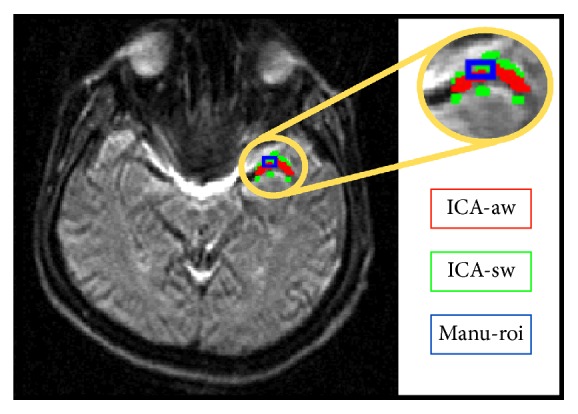
Regions selected by the manual ROI and ICA method (blue: manual selection; red and green: artery and its surrounding tissue selected by weighted-ICA).

**Figure 4:**
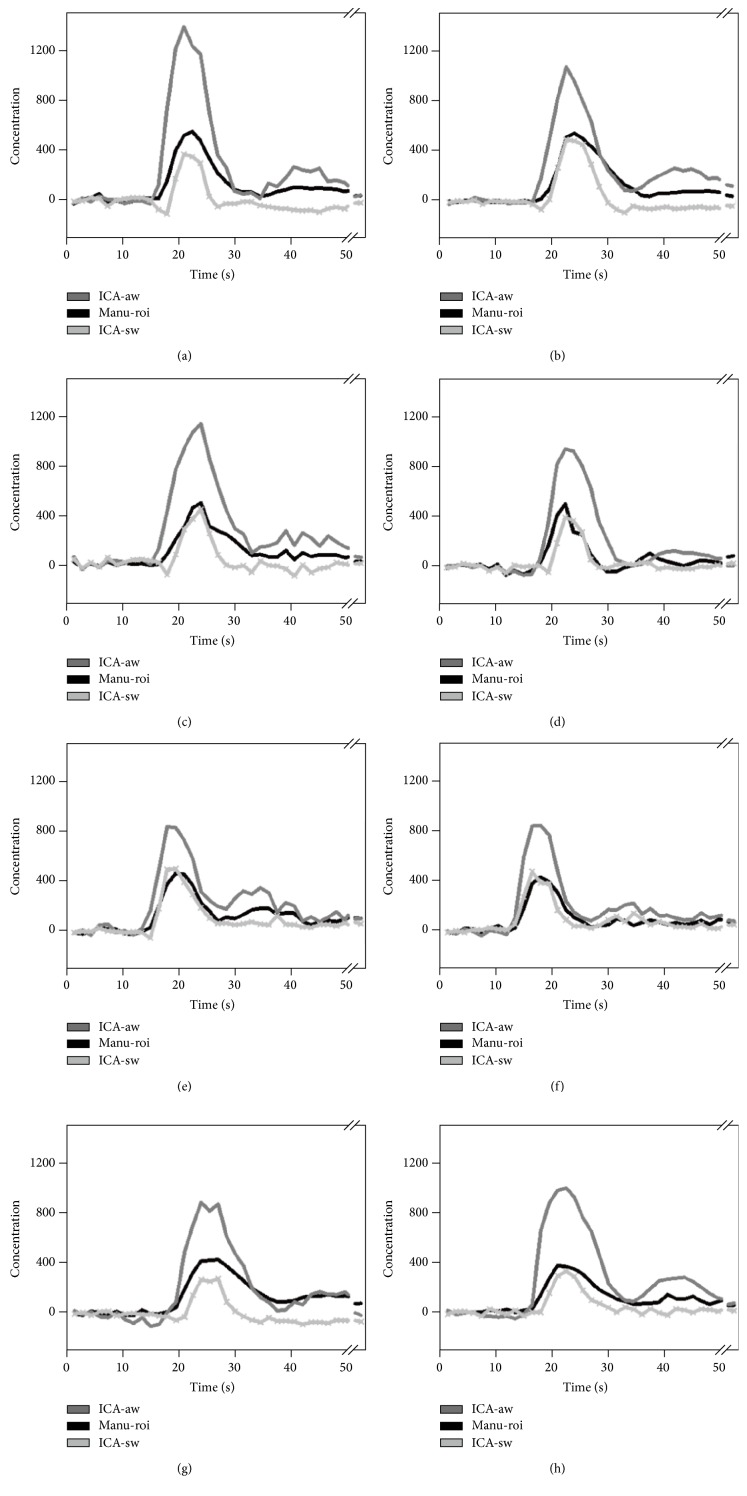
Different AIFs were determined for eight subjects in (a)–(h). (1) The artery with weighted-ICA (solid dark gray line); (2) the average with manual ROI (solid black line); and (3) the surrounding tissue with weighted-ICA (solid light gray line). This figure also shows the result.

Figure 3 shows the resultant segmentations at three regions for one clinical dataset: a region drawn manually by the manual ROI method and two regions segmented by the ICA method. It was found that ICA yielded better segmented boundaries along the MCA compared with the manual ROI, especially at the regions where the partial volume effect prevailed. In addition, the AIF concentration time curves for all subjects obtained from different methods were presented in Figure 4. As can be seen, the dynamic curve of the selected ROI showed its own tissue characteristic. The artery curve (solid dark gray line) had greater amplitude than the others and there was a clear recirculation after the first bolus passage. The surrounding tissue curve (solid light gray line) showed a lower peak than both the artery curve and the curve obtained from the manual ROI method (black line). Moreover, the manual ROI curve showed an intermediate characteristic between the above two. Although characteristics of AIF vary among subjects in onset time, peak height, FWHM and TTP, a consistent tendency among three AIFs determined by the different tissue location was consistent across subjects.

The results of AIF concentration time curves fitted with gamma-variate function for all eight subjects were listed in Table 1. The table showed the statistics of paired *t*-test between (a) manual ROI (Manu-roi) and the artery ROI with the weighted-ICA method (ICA-aw); (b) manual ROI (Manu-roi) and the surrounding tissue ROI with the weighted-ICA method (ICA-sw); and (c) artery ROI (ICA-aw) and the surrounding tissue ROI (ICA-sw) with the weighted-ICA method. Significant differences in peak height were found in various tissues, with the highest value in the artery and the lowest value in the surrounding tissue. A similar significant pattern was observed in arrival time, with the fastest for the artery and the slowest for the surrounding tissue. In addition, the TTP for the manual ROI was found to be longer than that for the other two methods while the FWHM for the surrounding tissue is smaller than that for the other two methods.

Table 2 showed the CBF values of gray and white matters and their ratios, obtained using AIFs calculated from the ROI selected by three methods. CBF values obtained from the AIF_w_ of surrounding tissue were significantly smaller than those obtained from the other two datasets. The CBF values calculated by the weighted-ICA method (ICA-sw) were 41.1 ± 4.9 and 22.1 ± 2.3 mL/100 g/min for cortical gray matter (GM) and deep white matter (WM) regions, respectively. The CBF values obtained from the manual ROIs were 53.6 ± 12.0 and 27.9 ± 5.9 mL/100 g/min for the same two regions, respectively. The CBF values and GM/WM ratios obtained from both methods (ICA and manual ROI) were in good agreement with those found in the literature [9].

## 4. Discussion

The partial volume effect is a common problem in the segmentation of the AIF location. It is almost impossible to select a region surrounding the arteries without partial volume confounding. However, the data-driven method provides a subjective approach to selecting the region of interest with characteristic information that can be used to avoid the partial volume effect. As the method used in this present study, ICA is a helpful tool and its applications are emerging in decades [31, 32]. Kao et al. [27] employed ICA aided by Bayesian estimation to segment the artery and to refine tissue classification. Other methods, for example, Murase et al. [18], utilized a fuzzy clustering method to identify the voxels belonging to the tissue around the artery after manually outlining the region. Even though the partial volume information is unknown from* a priori* knowledge, the relative fraction of the partial volume can be assessed by ICA processing. For the performance as shown in our simulation (in Figure 2), a good segmenting control was achieved in the region around the artery by weighting IC values. Then, the region possessing high source characteristics can be extracted by fraction thresholding of the partial volume. In a similar outcome also with respect to the clinical case in Figure 3, the regions selected from different locations were capable of determining a candidate AIF. The manual ROI method was more prone to the partial volume effect than the ICA when examining the surrounding tissue. It led to substantial uncertainty and required professional training to select an ROI for AIF determination.

As for ICA's specificity, ICA utilizes the spatial independence of constituent sources attributed to each voxel to decompose signals. These constituent sources include vessel components, tissue components, motion artificial components, and noise components induced by the contrast agent. For sources with more negentropy property (non-Gaussian), the ICA generates component maps with higher discrimination of the source signal [26, 27]. From the component maps, the source map of interest (i.e., vessel or tissue) is selected. Furthermore, the area confounded with higher partial volume effect can be removed by *z*-thresholding and weighting processing for the map of interest. ICA provides a good segmentation tool for locating the area of signal specificity as demonstrated in the simulation experiment and MRI perfusion data. Although ICA provides a better signal decomposition for tissue characteristics, its resultant constitution was normalized based on the hypothesis of statistic independence. This essential hypothesis restricted its application on the calculation of absolute partial volume in each brain tissue. However, if the density of brain parenchyma is involved in the treatment of disease, especially for the assessment of medication, an absolute estimation of tissue characteristics is worth further studies.

Except for the processor of data analysis, the signal we acquired can represent that the meaningful perfusion signal is not acquired easily because generally the signal is caused due to the tissue susceptibility and hemodynamic response. Therefore, according to the theoretical derivation of DSC-MR perfusion application, AIF derived from the region within the artery may not be a good choice because the relaxivities of arterial and tissue water differ. Previous studies assumed the same relaxivities for blood and tissue, which resulted in the overestimation of CBF in a nonlinear relation with the concentration in blood [30, 34]. Kiselev et al. suggested that the relaxivity of tissue should consider the contribution of both the static dephasing regime (SDR) and diffusional narrowing regime (DNR) effects [34–36]. The relaxation effect of Gd-DTPA in brain tissue was found to be several-fold larger than that in bulk blood [36]. Consequently, using AIF obtained from blood signals would introduce significant errors in quantifying CBF in brain tissue, unless the relaxivity of tissue could be independently measured. Besides the location at which the AIF needed to be determined, several properties of AIF were also in addition assessed for the comparison of CBF calculated among tissue in our present work. In Table 1, the manual ROI method was found to produce AIFs with different TTP, arrival time, FWHM, and peak height. These feature indexes revealed some biophysical properties of the contrast agent-affected compartments in the artery, surrounding tissue, and the region between them. The within-subject variation was mainly caused by the partial volume of tissue and blood. In addition, for the manual ROI method, the uncertainty of ROI selection (i.e., operating at different time sessions or by different operators) could lead to more variable results. This circumstance should be avoided in the application of study and clinical practice.

**Figure 5:**
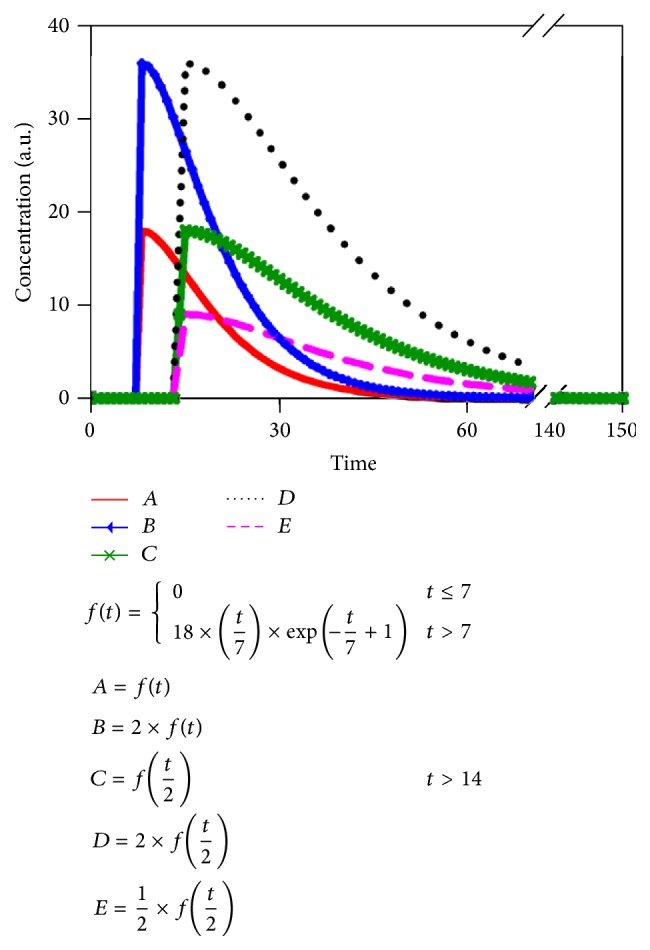
Various AIFs (*A*–*E*) are employed to test the deconvolution calculation for flow in dilution theory. “*A*” (red line) is the ideal AIF defined in the paper [38] and the ideal flow is 80 mL/100 g·min. The thresholding value is the cut-off value in adaptive SVD calculation [33]. *B*–*E* curves are the nonideal AIFs simulated.

In addition, a notion worth considering in the deconvolution process was that both the various shapes of the AIF and the thresholding in SVD processing could result in different CBF values. This effect was demonstrated by computer simulation using AIFs with various heights and areas (see Figure 5). In conclusion, using the nonideal AIFs will induce a large error in CBF estimation (Table 3). Next to the calculation of CBF, in this study, we also considered the averaged-AIF as the reference base. Although the comparison of averaged-AIF and weighted-AIF showed no significant difference to each other, it provided cross-checking of the intracorrelation evaluation (not shown averaged result). The top 50 voxels with highest *z*-value were highly consistent with each other and provided a partial volume free in the region.

In conclusion, quantitative CBF measurement involves a complex combination between tissue physiology, hemodynamic physics, data acquisition technique, and data analysis [37]. In the present work, we focused on the exploration of data mining, especially for the avoidance of partial volume effect. The benefits of this work were contributed for a more precise calculation of CBF: (1) ICA provides a semiautomatic tool for selecting the component of interest; (2) ICA decomposes the signal by reducing the partial volume effect; (3) the determination of AIF in the tissue around the artery is necessary for CBF quantification.

## Figures and Tables

**Table 1:** Comparisons of time-to-peak (TTP), arrival time (the first time point above the mean time response), FWHM, and peak high after gamma fitting in 8 subjects as determined by manual ROI (Manu-roi) and ICA in artery (ICA-aw) and surrounding tissue (ICA-sw). Below the table, there is statistic comparison with paired *t*-test for manual and weighted-ICA ROI. Significant difference between Manu-roi and ICA-aw ROI is found in TTP, onset time, and peak high and that between Manu-roi and ICA-sw ROI is found in TTP, onset time, FWHM, and peak. For ICA-aw and ICA-sw, there are significant differences in onset time, FWHM, and peak high.

	TTP (sec)			Onset time (sec)			FWHM (sec)			Peak high (#/mL)		
	Manu-roi	ICA-aw	ICA-sw	Manu-roi	ICA-aw	ICA-sw	Manu-roi	ICA-aw	ICA-sw	Manu-roi	ICA-aw	ICA-sw
#1 (36 y, m)	26.70	25.89	26.07	21.12	19.64	21.89	7.650	7.305	5.775	546.6	1423	324.4
#2 (33 y, m)	29.36	27.66	27.98	20.94	20.55	21.68	9.615	8.295	7.365	528.4	1020	456.5
#3 (32 y, m)	28.20	27.20	27.27	21.09	20.01	22.67	9.450	9.375	7.005	421.6	1077	337.3
#4 (46 y, m)	26.58	27.81	27.50	21.42	20.97	22.37	6.495	7.830	6.885	401.4	960.2	305.9
#5 (35 y, f)	24.74	24.18	24.03	18.68	18.60	19.08	9.060	8.655	6.090	427.8	792.2	508.3
#6 (35 y, f)	22.69	22.16	21.71	18.24	17.48	17.67	6.960	7.515	5.865	438.0	671.2	463.1
#7 (38 y, f)	30.50	29.41	29.58	22.85	22.88	25.73	12.53	9.240	4.920	422.3	903.7	309.9
#8 (29 y, f)	27.25	26.42	26.37	20.15	19.00	22.37	11.12	9.855	4.380	361.2	1033	411.1

Mean	27.00	26.34^a^	26.31^a^	20.56	19.89^a^	21.68^a,b^	9.110	8.509	6.036^a,b^	443.4	985.0	389.6^a,b^

Std^c^	2.487	2.281	2.456	1.503	1.642	2.427	2.060	0.931	1.036	62.78	222.6	79.98

^a^Significantly (*P* < 0.05, paired *t*-test) higher than Manu-roi.

^b^Significantly (*P* < 0.05, paired *t*-test) higher than ICA-aw.

^c^Standard deviation.

**Table 2:** rCBFs are calculated by the AIFs determined by the manual ROI (Manu-roi) and ICA-based ROI (weighted-ICA) method in gray and white matter regions. The paired *t*-test is employed to test the difference between the manual and ICA-based method in the artery (GM_*a*_ and WM_*a*_) and surrounding tissue (GM_*s*_ and WM_*s*_) and between the artery and surrounding tissue in the ICA-based method in gray and white matter regions.

rCBF (mL/100 g/min)	Manu-roi			ICA-aw			ICA-sw		
	GM_*m*_	WM_*m*_	(G/W)_*m*_	GM_*a*_	WM_*a*_	(G/W)_*a*_	GM_*s*_	WM_*s*_	(G/W)_*s*_
#1 (36 y, m)	66.98	38.35	1.746	56.13	35.17	1.596	41.94	24.83	1.689
#2 (33 y, m)	54.22	28.22	1.921	54.25	30.78	1.762	40.83	21.98	1.857
#3 (32 y, m)	42.26	24.05	1.757	48.00	30.60	1.569	35.02	21.14	1.656
#4 (46 y, m)	45.79	29.57	1.549	40.33	30.16	1.337	38.70	24.55	1.576
#5 (35 y, f)	64.09	28.75	2.229	60.73	28.78	2.110	46.52	23.68	1.964
#6 (35 y, f)	49.08	21.95	2.236	48.09	22.13	2.173	46.75	21.17	2.208
#7 (38 y, f)	37.33	20.13	1.855	41.74	24.73	1.688	34.05	17.71	1.922
#8 (29 y, f)	69.27	32.20	2.151	66.52	34.73	1.915	45.10	21.55	2.093

Mean	53.63	27.90	1.931	51.97	29.64	1.769^a^	41.11^a,b^	22.08^a,b^	1.871^a,b^

Std^c^	12.01	5.879	0.253	9.118	4.476	0.2839	4.935	2.309	0.221

GM = gray matter; WM = white matter; G/W = the ratio of gray matter and white matter.

^a^Significantly (*P* < 0.05, paired *t*-test) higher than ROI_*m*_ in GM, WM, and G/W.

^b^Significantly (*P* < 0.05, paired *t*-test) higher than *A*_wic_ in GM, WM, and G/W.

^c^Standard deviation.

**Table 3:** Estimated flow obtained from calculation of Figure 5.

	Estimated flow	Area under curve	Thresholding (%)
*A*	76.40	262.43	13.64
*B*	38.20	524.86	27.27
*C*	84.73	523.18	25.82
*D*	42.36	1046.36	51.64
*E*	168.91	262.43	12.95

Note: the signal-to-noise ratio is 27.7 for *A*.
